# Study of the IgG endoglycosidase EndoS in group A streptococcal phagocyte resistance and virulence

**DOI:** 10.1186/1471-2180-11-120

**Published:** 2011-05-27

**Authors:** Jonathan Sjögren, Cheryl YM Okumura, Mattias Collin, Victor Nizet, Andrew Hollands

**Affiliations:** 1 Division of Infection Medicine, Department of Clinical Sciences, Lund University, SE-221 84 Lund, Sweden; 2 Department of Pediatrics, University of California San Diego, 9500 Gilman Drive, Mail Code 0687, La Jolla, CA 92093, USA; 3 Skaggs School of Pharmacy and Pharmaceutical Sciences, University of California San Diego, 9500 Gilman Drive, Mail Code 0687, La Jolla, CA 92093, USA; 4 School of Biological Sciences, University of Wollongong, Wollongong, New South Wales, Australia

## Abstract

**Background:**

The secreted enzyme EndoS, an endoglycosidase from *Streptococcus pyogenes*, hydrolyzes the *N*-linked glycan of the constant region of immunoglobulin G (IgG) heavy chain and renders the antibody unable to interact with Fc receptors and elicit effector functions. In this study we couple targeted allelic replacement mutagenesis and heterologous expression to elucidate the contribution of EndoS to group A *Streptococcus *(GAS) phagocyte resistance and pathogenicity *in vitro *and *in vivo*.

**Results:**

Knocking out the EndoS gene in GAS M1T1 background revealed no significant differences in bacterial survival in immune cell killing assays or in a systemic mouse model of infection. However, exogenous addition and heterologous expression of EndoS was found to increase GAS resistance to killing by neutrophils and monocytes *in vitro*. Additionally, heterologous expression of EndoS in M49 GAS increased mouse virulence *in vivo*.

**Conclusions:**

We conclude that in a highly virulent M1T1 background, EndoS has no significant impact on GAS phagocyte resistance and pathogenicity. However, local accumulation or high levels of expression of EndoS in certain GAS strains may contribute to virulence.

## Background

Group A *Streptococcus *(GAS, *S. pyogenes*) is a human-specific pathogen producing diseases ranging from pharyngitis and impetigo to severe, invasive conditions such as necrotizing fasciitis and streptococcal toxic shock syndrome [1]. Causing an estimated 500,000 deaths annually [2], GAS is one of the world's most important pathogens, reflecting its wide repertoire of virulence factors that interfere with host immune clearance mechanisms [3]. A hypothesized GAS immune evasion factor is the secreted enzyme EndoS, an endoglycosidase possessing a highly specific hydrolyzing activity toward the *N*-linked glycan of immunoglobulin G (IgG) [4]. The IgG heavy chain is *N*-glycosylated at asparagine 297 with a complex biantennary oligosaccharide that is crucial for the interaction with Fc gamma receptors (FcγRs) on phagocytic cells [5-7]. Experimentally, enzymatic deglycosylation of murine IgG can decrease complement activation, binding of IgG to FcγRs on macrophages, and antibody-mediated cytotoxicity [5].

EndoS is specific to native IgG, which is in contrast to many related endoglycosidases that requires denaturation of their glycoprotein substrates [8,9]. Furthermore, pretreatment of IgG with recombinant EndoS diminishes its ability to opsonize bacteria and interact with FcγRs on leukocytes [10,11]. The activity of EndoS on IgG heavy chain glycans is well characterized and conserved among GAS serotypes [12]. However, a potential role of endogenous EndoS expression by the GAS bacterium in phagocyte resistance and virulence has not been elucidated. We hypothesize that EndoS contributes to GAS virulence by hydrolyzing the *N-*linked glycan on IgG and thereby impairing antibody mediated functions in the immune system. Here we couple targeted allelic replacement mutagenesis and heterologous gene expression to study EndoS activity during bacterial-host cell interaction *in vitro *and *in vivo*.

## Results

### Generation of EndoS mutants and heterologous expression

**Figure 1:**
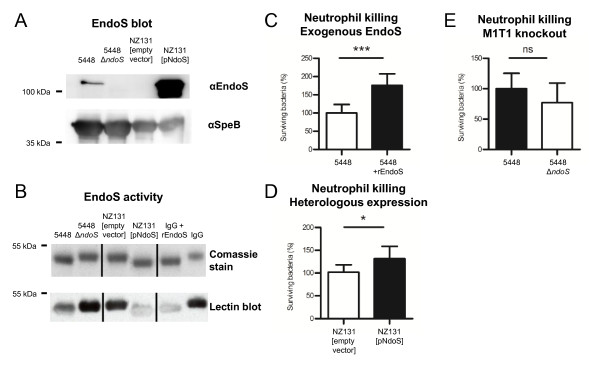
**EndoS expression and activity, and neutrophil killing assays**. (A) Western immunoblot showing EndoS expression in bacterial supernatants. SpeB is shown as a loading control. (B) Lectin blot analysis of murine IgG incubated with bacterial supernatants or rEndoS as a positive control. Opsonized bacterial survival in the presence of human neutrophils: (C) M1T1 GAS strain 5448 and isogenic *ndoS *knockout, 5448Δ*ndoS*. (D) Exogenous treatment of plasma with rEndoS prior to opsonization of GAS. (E) Heterologous expression of EndoS in NZ131 (serotype M49). Error bars indicate standard deviation from the mean. Experiments were performed in triplicate. * indicates *P *< 0.05, *** indicates *P *< 0.001, ns indicates no significant difference.

To investigate the contribution of EndoS to GAS and host-cell interactions an allelic replacement knockout in the M1T1 background was constructed and denoted 5448 Δ*ndoS*. Heterologous expression of EndoS in a non-native EndoS producing GAS strain, NZ131 (serotype M49), was established by transformation of the EndoS expressing plasmid pNdoS. Loss- and gain-of-function was confirmed by Western immunoblot (Figure 1A) and IgG glycan hydrolysis assays (Figure 1B) [8]. As suspected no detectable EndoS was identified in the supernatants of the 5448Δ*ndoS *strain, and heterologous expression of EndoS in NZ131 was successful. In addition, higher levels of EndoS were observed in the overexpressing strain NZ131 [pNdoS] compared to the wild-type M1 strain 5448.

### Neutrophil killing assay

The phagocytic resistance of GAS with and without EndoS contribution was investigated in a human neutrophil killing assay with GAS strains 5448Δ*ndoS *and wild-type 5448. Loss-of-function did not reveal significant difference in GAS resistance to phagocyte killing in the M1T1 background (Figure 1C). In the same M1T1 background, exogenous recombinant EndoS, rEndoS, or PBS was used to pretreat plasma to investigate phagocytic resistance contribution of the enzyme itself. It was found that rEndoS increases GAS survival in the presence of neutrophils and plasma containing GAS antibodies (Figure 1D). The contribution of EndoS to GAS virulence was also studied in the less virulent strain NZ131 (serotype M49) in gain-of-function analysis. The results reveal that heterologous overexpression of EndoS in M49, NZ131[pNdoS] increased GAS resistance to killing by human neutrophils (Figure 1E).

### Monocyte killing assay

**Figure 2:**
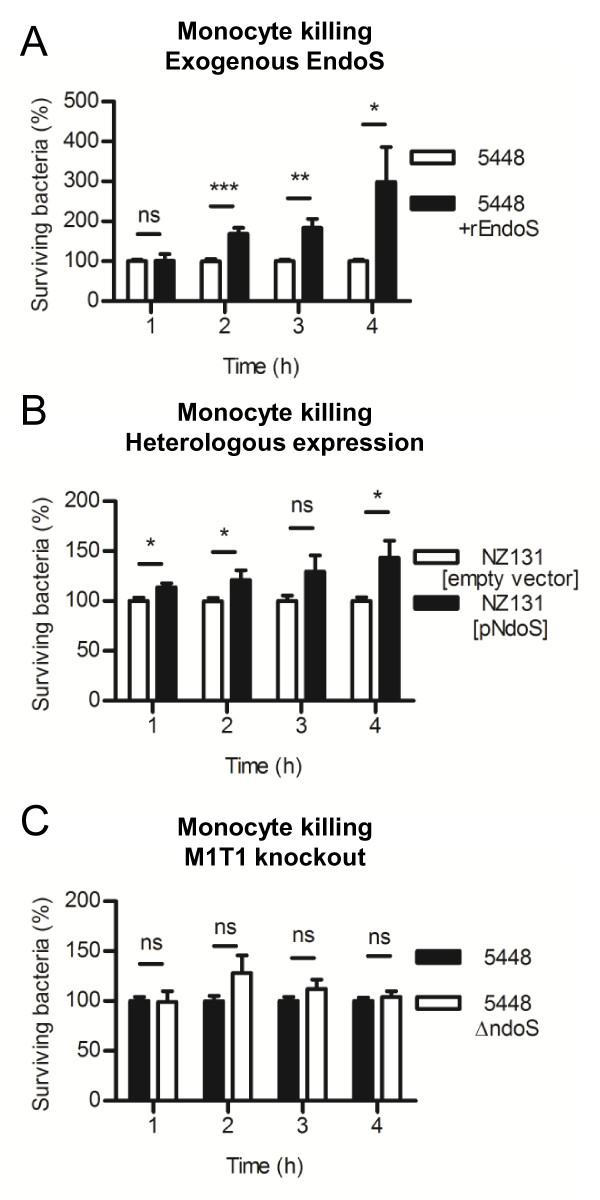
**Opsonized bacterial survival in U937 monocytic cell killing assays**. (A) M1T1 GAS strain 5448 and isogenic *ndoS *knockout, 5448Δ*ndoS*. (B) Exogenous pretreatment of plasma with rEndoS prior opsonization of GAS. (C) Heterologous expression of EndoS in NZ131 (serotype M49). Error bars indicate standard deviation from the mean. * indicates *P *< 0.05, ** indicates *P *< 0.01, *** indicates *P *< 0.001, ns indicates no significant difference.

As with neutrophil killing assays, no significant difference in bacterial survival was detected in the monocytic killing assays when comparing M1T1 GAS strain 5448 to the isogenic *ndoS *knockout strain (Figure 2A). Pretreatment of plasma with exogenous rEndoS resulted in a significant increase in GAS resistance to killing by monocytes (Figure 2B), as did heterologous expression of EndoS in the less virulent strain NZ131 (Figure 2C).

### *In vivo *mouse model

**Figure 3:**
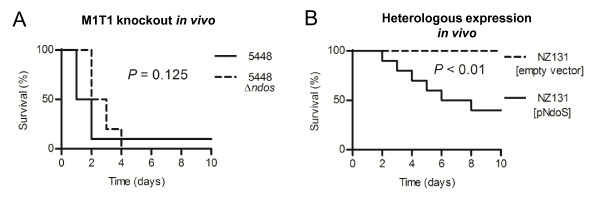
**Survival curves of female CD-1 mice following intraperitoneal challenge with GAS**. (A) M1T1 GAS strain 5448 and isogenic *ndoS *knockout, 5448Δ*ndoS*, at 2 × 10^7 ^cfu with 5% mucin (n = 6). (B) Heterologous expression of EndoS in NZ131 (serotype M49) at 5 × 10^8 ^cfu with 5% mucin (n = 10).

Many major GAS virulence factors have been shown to decrease overall virulence when knocked out and studied in murine infection models [13-16]. It has also been shown that EndoS has activity on all subclasses of murine IgG [17]. Taken together, this led us to believe that the contribution of EndoS to GAS virulence could be studied *in vivo*. However, in this murine model of infection GAS strain 5448Δ*ndoS *showed no significant difference in virulence compared to wild-type 5448 (Figure 3A).

However, when we studied the less virulent GAS strain NZ131 (serotype M49) overexpressing EndoS, it was found that strain NZ131[pNdoS] showed increased virulence *in vivo *(Figure 3B) compared to wild-type NZ131[empty vector]. This may be a function of the relatively high level of expression of EndoS in NZ131[pNdoS] compared to 5448 (Figure 1A).

## Discussion

A single clone of the M1T1 serotype has disseminated globally during the last few decades to represent the leading cause of severe, invasive GAS infections [18]. The unique virulence of the M1T1 clone has been associated with many factors including the phage-encoded DNAse Sda1, allowing escape from neutrophil extracellular traps [13,19,20], the streptococcal inhibitor of complement (SIC) protein, promoting serum and antimicrobial peptide resistance [14,21], pro-inflammatory and phagocyte resistance properties of the M1T1 protein [15,22], high level expression of the pore-forming cytotoxin streptolysin O (SLO) [16], and a propensity for genetic mutations in the *covR/S *regulatory locus promoting hypervirulence [23,24]. There exist many inherent limitations of modeling a secreted bacterial virulence factor *in vitro *and of the mouse as a surrogate host for GAS infection studies. However, our studies do strongly suggest that the endogenous expression of EndoS may be redundant or dispensable for M1T1 GAS phagocyte resistance and pathogenicity, since targeted mutation of the other factors described above do yield clear attenuation of virulence phenotypes in similar *in vitro *and *in vivo *assay systems.

Conversely, pretreatment of plasma containing antibodies against GAS with recombinant EndoS reduced opsonphagocytic killing of GAS, and heterologous overexpression of EndoS in a less virulent M49 GAS strain conferred increased phagocyte resistance and increased lethality in the mouse infection model. These results suggest that high level expression or local accumulation of EndoS in tissues could contribute to virulence in certain GAS strain backgrounds or infection scenarios, a subject that could merit future analysis in larger clinical or molecular epidemiologic surveys.

EndoS is highly conserved among GAS serotypes and can also be found in *Streptococcus equi *and *zooepidemicus *[12]. Therefore, it was somewhat surprising that we could not detect a significant contribution to GAS virulence *in vivo*. This may be due to the limitations of the mouse model used, and the expression levels of EndoS during the murine infection. The expression level of this enzyme during a human infection could have an impact on GAS immune cell killing resistance but this remains to be investigated. The specificity of EndoS activity towards IgG suggests that the enzyme may have an important role in the pathogenesis of GAS, yet to be discovered.

Finally, whether or not GAS can effectively deploy this unique enzymatic activity targeted IgG *N*-glycosylation to promote its own survival in the host (as is intuitively appealing), the enzyme itself has already proven a promising lead biotherapeutic for treatment of antibody-mediated inflammatory pathologies [17,25-29].

## Conclusions

We conclude that in a highly virulent M1T1 background, EndoS has no significant impact on GAS phagocyte resistance and pathogenicity. However, our overexpression experiments could indicate that local accumulation or high levels of expression of EndoS can contribute to virulence in certain GAS strains, or in other infection scenarios than the systemic infection model used in this study.

## Methods

### Bacterial strains and growth

GAS strain 5448 (serotype M1T1, *ndoS-*positive) and GAS strain NZ131 (serotype M49, *ndoS-*negative) are well-characterized and were selected for use in this study [30,31]. *Escherichia coli *MC1061 was used as cloning tool [32]. The streptococcal and *E. coli *strains were propagated on Todd-Hewitt agar (THA). For selection, erythromycin (*erm*) was used at 5 μg/mL (5448), 2 μg/mL (NZ131) and 500 μg/mL (MC1061). GAS and its isogenic mutant were grown in Todd-Hewitt broth (THB (Difco, Detroit, MI)) at 37°C without shaking. For *in vitro *and *in vivo *experiments, fresh overnight cultures were diluted 1:10 in THB and grown to mid logarithmic phase (OD_600 _= 0.4) and resuspended in PBS, or in mid-log supernatants for neutrophil assays with NZ131. For analysis of streptococcal supernatants, strains were grown in C-medium (0.5% (w/v) Proteose Peptone no. 2 (Difco), 1.5% (w/v) yeast extract, 10 mM K_2_HPO_4_, 0.4 mM MgSO_4_, 17 mM NaCl pH 7.5) to maximize EndoS expression.

### GAS mutants

EndoS is encoded by the gene *ndoS*. A precise, in-frame allelic replacement of *ndoS *with chloramphenicol transferase, *cat*, was created in M1T1 GAS strain 5448 by a method previously described [13] and was denoted 5448Δ*ndoS*. Briefly, a 798 bp fragment upstream, and 987 bp fragment downstream of *ndoS *was amplified using polymerase chain reaction, PCR, using primers *ndoS*-up-F-XbaI (GCATCTAGAGCTTGTCGGTCTTGGGGTAGC), ndoS-up-R (GGTGGTATATCCAGTGATTTTTTTCTCCATTTGGACACTCCTTATTTTTGGTACTAAGT C) and *ndoS*-dn-F (TACTGCGATGAGTGGCAGGGCGGGGCGTAAACAAAGTAACTTTCTTAGATAGCAACATT CAG), *ndoS*-dn-R-BamHI (GCGGATCCGTTCTTGCGCCATGACACCTCC) respectively. The primers adjacent to *ndoS *contained 30 bp overhang of the *cat *gene corresponding to the 5' and 3' ends of *cat*, respectively. The upstream and downstream fragments were combined with the 650 bp *cat *gene in a fusion PCR using primers *ndoS*-up-F-XbaI and *ndoS*-dn-R-BamHI. This triple fragment was digested using restriction enzymes *Xba*I and *BamH*I and ligated using T4 ligase into the temperature sensitive vector pHY304, bearing erythromycin resistance, to generate the knockout plasmid pHY-*ndoS*-KO. pHY-*ndoS*-KO was transformed into GAS 5448 by electroporation and transformants were grown at the permissive temperature of 30°C with erythromycin. Transformants were then grown at the non-permissive temperature of 37°C with erythromycin present to select for homologous recombination and integration of the plasmid into the genome. Single crossovers were confirmed by PCR analysis. Relaxation of the plasmid was carried out at 30°C with no antibiotic selection to allow the plasmid to reform, outside the chromosome. Growing the bacteria at 37°C without antibiotic pressure resulted in loss of the plasmid. Finally, screening for erythromycin sensitive colonies was used to identify double crossover events and allelic replacement mutants were confirmed by PCR. In frame allelic replacement *ndoS *mutant, 5448Δ*ndoS*, was confirmed by multiple PCR reactions showing the insertion of the *cat *gene and absence of the *ndoS *gene in the genome. Heterologous expression of EndoS in M49 GAS strain NZ131 was established by transformation with the EndoS expression plasmid pNdoS. *ndoS *was amplified from the M1 genome using primers *ndoS-*F-EcoRI (GCGAATTCATGGATAAACATTTGTTGGTAAAAAGAAC) and *ndoS-*R-BamHI (GCGGATCCTTATTTTTTTAGCAGCTGCCTTTTCTC), digested with *EcoR*I and *BamH*I prior to T4-ligation into the expression vector pDCerm, denoted pNdoS. As a control, GAS strain NZ131 was transformed with the empty vector pDCerm to generate NZ131[empty vector].

### Western blot

Supernatants from stationary phase (16 h) GAS strains 5448, 5448Δ*ndoS*, NZ131[empty vector] and NZ131[pNdoS] were precipitated with 5% final concentration of trichloroacetic acid and separated on a 10% SDS-PAGE gel and blotted onto a methanol activated PVDF membrane. The membrane was blocked in 5% skimmed milk (Difco) for 1 h and washed 3 × 10 minutes in phosphate buffered saline, PBS (137 mM NaCl, 2.7 M KCl, 10 mM Na_2_HPO_4_, 2 mM KH_2_PO_4_, pH 7.4). The membrane was then incubated with polyclonal rabbit antiserum against rEndoS at 1:2000 dilution in 0.5% skimmed milk and incubated for 1 h at 37°C. The membrane was washed as before and incubated with goat anti-rabbit IgG conjugated with Horse radish peroxidase (Bio-Rad), at 1:5,000 in 0.5% skimmed milk for 1 h at 37°C. After washing, the membrane was developed using Supersignal West Pico Chemiluminescent (Thermo Scientific, Rockford, IL) and analyzed on a Chemidoc XRS (Bio-Rad, Hercules, CA).

### Lectin blot

Supernatants from GAS strains 5448, 5448Δ*ndoS*, NZ131[empty vector] and NZ131[pNdoS] at stationary phase (16 h) was incubated with 1 μg murine IgG (mIgG) for 2 h at 37°C at static conditions. As a positive control, IgG was incubated with 1 μg rEndoS. The glycan hydrolyzing activity was analyzed with SDS-PAGE and lectin blot using biotinylated *Lens culinaris agglutinin *(LCA) (Vector Laboratories, Burlingame, CA). LCA lectin recognizes the α-1,3 mannose residue found on the *N-*linked glycan on IgG. Briefly, the supernatants and mIgG were separated on 10% SDS-PAGE gels, onestained with Coomassie blue and the other blotted onto Immobilon PVDF membranes (Millipore, Bedford, MA). The membrane was blocked in lectin buffer (10 mM HEPES, 0.15 M NaCl, 0,1% Tween 20, 0.01 mM MnCl_2_, 0.1 mM CaCl_2_, pH = 7.5) for 1 h. 10 μg LCA in lectin buffer was incubated with the membrane for 1 h at RT. The membrane was then washed for 3 × 10 min in lectin buffer and incubated with 2 μg streptavidin linked HRP (Vector Laboratories) for 1 h. After washing as above the blot was developed using Supersignal West Pico Chemiluminescent (Thermo Scientific) as described for Western blots.

### Neutrophil killing assay

Neutrophils were purified from healthy donors using PolyMorphPrep-kit (Axis-Shield, Oslo, Norway) and RBCs lysed with sterile H_2_0 as previously described [33]. Neutrophils were seeded at 2 × 10^5 ^cells/well in 96-well microtiter plates in RPMI.

Plasma was obtained from healthy volunteers as previously described [33]. All neutrophil and plasma donors exhibited high serum titer (>1:20,000) against serotype M1 and M49 GAS (Additional file 1 Table S1). GAS strains were grown as described and opsonized for 1 h at 37°C in 80% plasma, with or without pretreatment using recombinant EndoS (rEndoS) under rotating conditions. For pretreatment, 1 mL of plasma was incubated with 50 μg of rEndoS or PBS (control) at 37°C for 2 h with rotation. The bacteria were then diluted to the desired concentration in RPMI with a final concentration of 2% plasma and added to the neutrophils at a multiplicity of infection (MOI) of 10 bacteria per cell. Control wells contained GAS in RPMI and 2% plasma without neutrophils. The plate was centrifuged at 500 × *g *for 10 min and incubated for 30 min at 37°C with 5% CO_2 _before being serially diluted in sterile H_2_O and triplicate wells were plated on Todd-Hewitt agar (THA) plates for enumeration. Percent survival of the bacteria was calculated relative to control wells. Data from three separate experiments were normalized to 5448 or NZ131[empty vector] and combined.

### Monocyte killing assay

The human monocytic cell line U937 was seeded at 5 × 10^5 ^cells/well in RPMI supplemented with 10% fetal bovine serum (FBS) in 24-well plates. GAS was grown and pre-opsonized in human plasma with or without rEndoS treatment, as described above. Bacteria were grown as described above and added to the U937 cells at MOI = 10 and incubated at 37°C with 5% CO_2_. Samples were collected at 1, 2, 3 and 4 h when monocytes were lysed with 0.025% Triton X-100 (MP Biomedicals, Aurora, OH) and triturated vigorously. Surviving bacteria from triplicate wells were plated on THA for enumeration. Percentage of surviving bacteria was calculated relative to the initial innoculum. Data from at least three separate experiments were normalized to 5448 or NZ131[empty vector] and combined.

### Determination of donor serum titers

Blood from healthy human donors was collected in glass venous blood collection tubes with no additives (BD Biosciences, San Jose, CA) and clotted at room temperature for 15 min. Blood was centrifuged at 3,200 × g for 10 min at 4°C. The serum fraction was collected and stored at -80°C.

GAS strains NZ131 (serotype M49) and 5448 (serotype M1) were grown to mid-log phase in THB. Bacteria were resuspended in PBS and heat-killed at 95°C for 10 min. Heat-killed bacteria were mixed with a final concentration of 0.1 M NaHCO3 pH 9.6 and 10^6 ^bacteria per well were coated to 96-well high-bind ELISA plates (Costar, Cambridge, MA) at 4°C overnight. Plates were washed with PBS + 0.05% Tween (PBS-T) and blocked with 4% BSA + 10% FBS in PBS-T for 1 h at 37°C. Serum samples were diluted in blocking solution and incubated for 2 h at 37°C. Plates were washed with PBS-T and incubated with 1:5000 dilution of HRP-conjugated goat anti-human IgG antibody (Promega, Madison, WI) for 1 h at room temperature. Plates were washed five times with PBS-T and incubated with TMB substrate reagent (BD OptEIA TMB Substrate Reagent Set, BD Biosciences) at room temperature for 30 min. The reaction was stopped with an equal volume of 0.2 N sulfuric acid, and the plate was read at 450 nm. End point titer was determined as the dilution giving signal above a calculation cutoff determined using a mouse serum negative control and the calculation method described in [34].

### *In vivo *mouse model

To evaluate the contribution of EndoS to GAS virulence *in vivo*, we utilized a murine model of systemic infection. GAS strains were grown as described and resuspended in PBS with 5% mucin for an inoculum of 2 × 10^7 ^cfu for WT M1T1 strain 5448 and isogenic mutant 5448Δ*ndoS*, and 5 × 10^8 ^cfu for NZ131[empty vector] and NZ131[pNdoS]. 8-10 week old female CD-1 mice (n = 6 for 5448, n = 10 for NZ131) were infected intraperitoneally with GAS strains and mortality was monitored daily for 10 days.

### Statistical analysis

Cfu enumeration in neutrophil and monocyte killing assays were statistically analyzed by unpaired Student's *t*-test. Differences were considered significant if *P <*0.05. The *in vivo *results were evaluated with log-rank (Mantel-Cox) test for comparison of survival curves. Differences in survival were considered significant if *P *< 0.05. All statistical analysis was performed using GraphPad Prism v.5 (GraphPad Software).

### Ethical approval

Permission to collect human blood under informed consent was approved by the UCSD Human Research Protections Program. All animal use and procedures were approved by the UCSD Institutional Animal Care and Use Committee.

## Conflicts of interests

Patents for the *in vitro *and *in vivo *use of EndoS have been applied for by Genovis AB and Hansa Medical AB, respectively. MC is listed as inventor on these applications that are pending. Hansa Medical AB in part funded this study, but had no influence on the design of study, interpretation of data, or the final form of the manuscript. MC is a part time scientific consultant for Hansa Medical AB.

## Authors' contributions

JS participated in the design of the study, performed experiments and drafted the manuscript. MC and VN conceived of the study. CO performed experiments. AH designed the study and performed experiments. All authors read and approved the final manuscript.

## Supplementary Material

Additional file  1 **Table S1**. Click here for file
