# Supplementary material for: Study of the IgG endoglycosidase EndoS in group A streptococcal phagocyte resistance and virulence
Source: BMC Microbiol. 2011 May 27;11:120. doi: 10.1186/1471-2180-11-120 (PMC3125321; doi:10.1186/1471-2180-11-120)
Supplement: Additional file 1 — Table S1. [file 1471-2180-11-120-S1.PDF]

Table S1: GAS serum titers of donors

Serum titer against heat-killed GAS (serotype M1 and M49) was determined for all donors whose plasma was used in neutrophil (Fig 1) and monocyte (Fig 2) killing assays.

Donor	M1 (strain 5448) titer	M49 (strain NZ131) titer
1	1:100,000	1:80,000
2	1:200,000	1:200,000
3	1:200,000	1:100,000
4	1:100,000	1:50,000
5	>1:500,000	1:200,000
6	1:20,000	1:20,000
